# Lack of acquired resistance in HER2-positive breast cancer cells after long-term HER2 siRNA nanoparticle treatment

**DOI:** 10.1371/journal.pone.0198141

**Published:** 2018-06-07

**Authors:** Shenda Gu, Worapol Ngamcherdtrakul, Moataz Reda, Zhi Hu, Joe W. Gray, Wassana Yantasee

**Affiliations:** 1 Department of Biomedical Engineering, Oregon Health & Science University, Portland, Oregon, United States of America; 2 PDX Pharmaceuticals, LLC, Portland, Oregon, United States of America; Northern University, UNITED STATES

## Abstract

Intrinsic and acquired resistance to current HER2 targeted therapies remains a challenge in clinics. We have developed a therapeutic HER2 siRNA delivered using mesoporous silica nanoparticles modified with polymers and conjugated with HER2 targeting antibodies. Our previous studies have shown that our HER2 siRNA nanoparticles could overcome intrinsic and acquired resistance to trastuzumab and lapatinib in HER2-positive breast cancers. In this study, we investigated the effect of long-term (7 months) treatment using our therapeutic HER2 siRNA. Even after the removal of HER2 siRNA, the long-term treated cells grew much slower (67% increase in doubling time) than cells that have not received any treatment. The treated cells did not undergo epithelial-mesenchymal transition or showed enrichment of tumor initiating cells. Unlike trastuzumab and lapatinib, which induced resistance in BT474 cells after 6 months of treatment, HER2 siRNA did not induce resistance to HER2 siRNA, trastuzumab, or lapatinib. HER2 ablation with HER2 siRNA prevented reactivation of HER2 signaling that was observed in cells resistant to lapatinib. Altogether, our results indicate that a HER2 siRNA based therapeutic provides a more durable inhibition of HER2 signaling in vitro and can potentially be more effective than the existing therapeutic monoclonal antibodies and small molecule inhibitors.

## Introduction

Overexpression or amplification of HER2 (*ERBB2*) occurs in several types of cancer including breast [1], ovarian [2, 3], gastric [4] and colorectal cancers [5, 6]. In breast cancer, approximately 20% of all cases fall into the HER2-positive subtype, which is an adverse prognosis factor [7]. Current therapeutic regimens include the use of monoclonal antibodies or small molecule inhibitors in combination with chemotherapy such as taxane. Approved in 1998, the humanized monoclonal antibody trastuzumab binds to domain IV of HER2 and blocks receptor homodimerization [8]. To complement the activity of trastuzumab, another monoclonal antibody, pertuzumab, was developed and it binds to domain II of HER2 to block heterodimerization with other HER family receptors [9]. Following positive results of the CLEOPATRA trial, these two antibodies are now used with docetaxel as first-line treatment for metastatic HER2-positive breast cancer [10, 11]. The recently approved trastuzumab emtansine (T-DM1) is trastuzumab conjugated to the microtubule inhibitor emtansine and is used as a second-line treatment [12]. Lapatinib, a small molecule inhibitor that binds to the kinase domain of HER2 and EGFR [13, 14], is used in combination with trastuzumab or capecitabine as a third-line treatment [15].

Despite the development and use of HER2 targeted therapeutics, intrinsic and acquired resistance remains a challenge in the clinics. For instance, in the phase 3 MARIANNE trial, 67.9% of patients responded to trastuzumab plus a taxane with a median response duration of 12.5 months, while 64.2% of patients responded to T-DM1 plus pertuzumab with a median response of 21.2 months [16]. Ongoing research into the mechanisms of resistance have revealed several molecular adaptations in which tumor cells can compensate for or circumvent the inhibition of HER2 signaling. Mutations in the kinase domain of HER2 that cause resistance to lapatinib have been characterized but are rare [17–19]. Truncation of the extracellular domain of HER2 produces a variant called p95HER2 that does not respond to monoclonal antibodies [20–22]. Upregulation of MUC4 can block binding of trastuzumab [23, 24]. There have been reports of Δ16HER2 in which exon 16 is excised during splicing, resulting in a form that homodimerizes more readily [25, 26]. In addition, cancer cells can overcome HER2 blockade by signaling through other HER family receptors [27–29] or receptor crosstalk via MUC1-C [30], IGFR [31], MET [32] and FGFR [33]. Furthermore, studies have shown that resistant cancer cells can reactivate the HER2 pathway via secretion of HER receptor ligands, and thus remain dependent on HER2 signaling [34, 35].

We sought to find alternative approaches to monoclonal antibodies and small molecule inhibitors to provide durable responses. We developed a pre-clinical HER2 siRNA based therapeutic delivered using functionalized mesoporous silica nanoparticles. The nanoparticles consist of 50-nm silica cores coated with a cationic polymer and PEG and conjugated to trastuzumab for HER2 targeting. We have previously shown that our HER2 siRNA nanotherapeutic has excellent safety profile and could overcome intrinsic and acquired resistance to trastuzumab and lapatinib in HER2-positive breast cancers *in vitro* and *in vivo* [36, 37]. In the current study, we investigate the response duration of cancer cells treated with HER2 siRNA delivered by our nanoparticles or the commercial transfection reagent DharmaFECT as benchmark. While DharmaFECT can be used in vitro to study the functional significance of long-term HER2 ablation, the ability of our nanoparticles to deliver siRNA *in vivo* has far more clinical relevance. We hypothesize that ablation of HER2 protein by siRNA can prevent the rapid onset of resistance. We compared how the cells differ in their response to trastuzumab, lapatinib or HER2 siRNA after long-term treatment to these drugs. We also explored changes in protein expression and phosphorylation using reverse phase protein arrays (RPPA) to determine the adaptive changes necessary to survive in a low HER2 environment. In all, our findings suggest that targeting HER2-positive cancer using siRNA can potentially be more durable and effective than monoclonal antibodies or small molecule inhibitors.

## Materials and methods

### Synthesis of nanoparticles and preparation of siRNA complexes

Mesoporous silica nanoparticles modified with polymer and conjugated to trastuzumab were synthesized and characterized as previously reported [37]. HER2 and non-targeting control siRNAs (siSCR) purchased from Dharmacon™ were loaded onto nanoparticles at 2 wt.% prior to transfection. For transfection using DharmaFECT, siRNAs were diluted in OptiMEM medium and a final dilution ratio of 1:200 was used for DharmaFECT.

### Cell culture and long-term treatment

BT474 was obtained from ATCC and maintained in RPMI1640 growth medium supplemented with 10% fetal bovine serum. BT474-TR and BT474-LR were generated by growing the parental BT474 under increasing concentrations of trastuzumab or lapatinib for 6 months, as previously reported [36]. For long-term siRNA transfection, cells were seeded in 6-well plates and transfected weekly with 60 nM HER2 siRNA or 60 nM scrambled siRNA delivered by nanoparticles or DharmaFECT.

### Cell viability assay

Cell viability was determined using the CellTiter-Glo assay (Promega). Cells were seeded in 96-well plates and allowed to attach for 24 h prior to transfection or drug treatment. Plates were read 3 days after lapatinib treatment or 5 days after trastuzumab treatment or siRNA transfection. For treatment with siRNAs, cell media were changed 24 hours after transfection.

### Flow cytometry

Freshly harvested cells were washed in FACS buffer (pH 7.4 PBS with 1 mM MgCl_2_, 0.1 mM CaCl_2_, 1% FBS and 0.02% sodium azide) and aliquoted into 1×10^6^ fractions for staining. CD24-FITC and CD44-APC antibodies (BD Biosciences) were added according to the manufacturer’s recommended dilution and the samples were incubated on ice with shaking for 30 min. After two washes, samples were resuspended in 500 μl of FACS buffer and analyzed on a Millipore Guava easyCyte 12 flow cytometer. HER2 was stained using 1 μg of trastuzumab per sample followed by washing and incubation with 1 μg of Alexa 647 conjugated anti-human secondary antibody.

### Western blot

Cells were lysed in RIPA buffer, sonicated and protein was quantified using BCA assay. After adding 4X Novex NuPAGE LDS sample buffer and 10% beta-mercaptoethanol (BME), the samples were denatured for 5 min at 95°C. Twenty to 30 μg of proteins were loaded per lane onto 4–12% Bis-Tris NuPAGE gels. Following gel electrophoresis, proteins were transferred onto PVDF-FL membrane, blocked with LI-COR blocking buffer and incubated with primary antibodies overnight at 4°C. IRDye conjugated secondary antibodies were added the next day and membranes were scanned on a LI-COR Odyssey CLx imaging system. Band densitometry was analyzed using ImageJ.

### Reverse phase protein microarray

Protein from the parental and long-term treated BT474 derivatives were harvested and processed according to the protocol from MD Anderson’s RPPA core facility website [38]. Briefly, cells in 6-well plates were lysed and proteins were collected. Debris were removed after centrifugation and proteins were quantified and adjusted to 1.5 μg/μl. SDS and BME were added and samples were denatured at 96°C for 5 min. Protein samples were stored at -80°C until dispatched to MD Anderson’s RPPA core facility for analysis. Detailed RPPA process and methods can be found on MD Anderson’s RPPA core facility website [39].

### Statistical analysis

Experiments were performed in three or more replicates with results reported as mean ± standard deviation (SD). Student t test (normal distribution) or Mann-Whitney test (nonparametric, unpaired) was used for group comparison. Multiple comparisons of three or more groups were done using one-way ANOVA (normal distribution) or the Kruskal-Wallis nonparametric test with post-hoc Dunnett multiple comparison tests. GraphPad Prism 6.0 software (GraphPad Software Inc.) was used for all statistical analyses. P < 0.05 was considered statistically significant.

## Results

We previously reported that BT474 cells became resistant to 10 μg/ml of trastuzumab and 1 μM of lapatinib within 6 months of continuous treatment [36]. To determine whether BT474 cells could develop resistance to HER2 siRNA, we performed similar long-term treatment by transfecting the cells weekly with HER2 siRNA delivered with either DharmaFECT or our mesoporous silica nanoparticles for 7 months. Nanoparticles with (TNP) and without trastuzumab (NP) were tested to account for any therapeutic effect of trastuzumab. Trastuzumab conjugated nanoparticles showed uptake specificity to HER2-positive cancer cells over HER2-negative cells after a short contact time of 30 minutes to 2 hours [37], while nanoparticles without trastuzumab (more positively charged) entered the cells in a non-specific manner after long exposure. Separate populations were treated with scrambled siRNAs (siSCR) for the same duration as another control. The resulting derivatives and treatment description are listed in Table 1, and their names will be used thereafter.

**Table 1:** Name and corresponding treatment of BT474 derivatives, all with 30 weeks of treatment.

Name	Treatment	Description
BT474-C30	siSCR	scrambled siRNA with DharmaFECT
BT474-H30	siHER2	HER2 siRNA with DharmaFECT
BT474-NP-C30	siSCR-NP	scrambled siRNA on nanoparticles
BT474-NP-H30	siHER2-NP	HER2 siRNA on nanoparticles
BT474-TNP-C30	T-siSCR-NP	scrambled siRNA on trastuzumab conjugated nanoparticles
BT474-TNP-H30	T-siHER2-NP	HER2 siRNA on trastuzumab conjugated nanoparticles
BT474-TR	Trastuzumab	Trastuzumab resistant BT474
BT474-LR	Lapatinib	Lapatinib resistant BT474

### Reduced cell cluster size and growth rate after long-term treatment with HER2 siRNA on nanoparticles conjugated to trastuzumab

**Fig 1:**
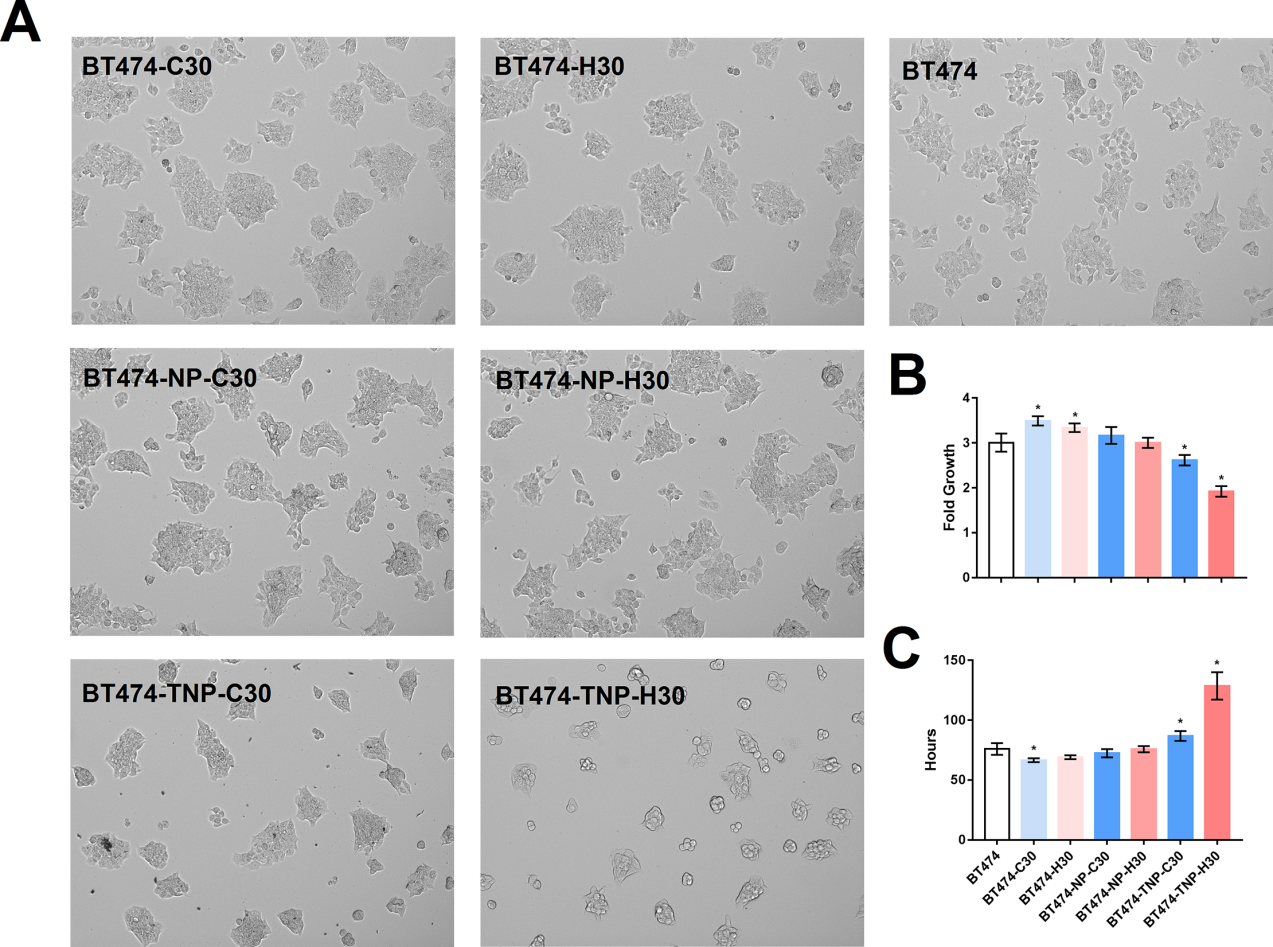
Phenotype of long-term siRNA treated cells. When seeded at the same density for 5 days without treatment, BT474-TNP-H30 grew slower than parental BT474 and other derivatives. Images showing representative fields at 100X magnification **(A)**. Fold change in nuclei count after 5 days of growth **(B)** and the corresponding doubling time **(C)**. Bars represent mean ± SD of 6 replicates in a 96-well plate. Asterisks * indicate statistical significance when compared to the parental BT474 (P < 0.05).

Fig 1A–1C show the cell morphology and growth rate of each BT474 derivatives after the long-term treatment. Cells were seeded at the same density 5 days before imaging or nuclei counting. BT474 treated with HER2 siRNA on nanoparticles conjugated with trastuzumab grew 30% slower than the parental or the scrambled siRNA treated counterparts (Fig 1B) with a doubling time that was almost twice as long (Fig 1C). Interestingly, we did not observe such change in cells treated with HER2 siRNA or scrambled siRNA delivered using DharmaFECT or nanoparticle without trastuzumab. This may owe to the increased cellular uptake and/or therapeutic effect of trastuzumab in addition to that of HER2 siRNA.

### Long-term HER2 siRNA treatment did not lead to epithelial-mesenchymal transition or tumor initiating cell enrichment

**Fig 2:**
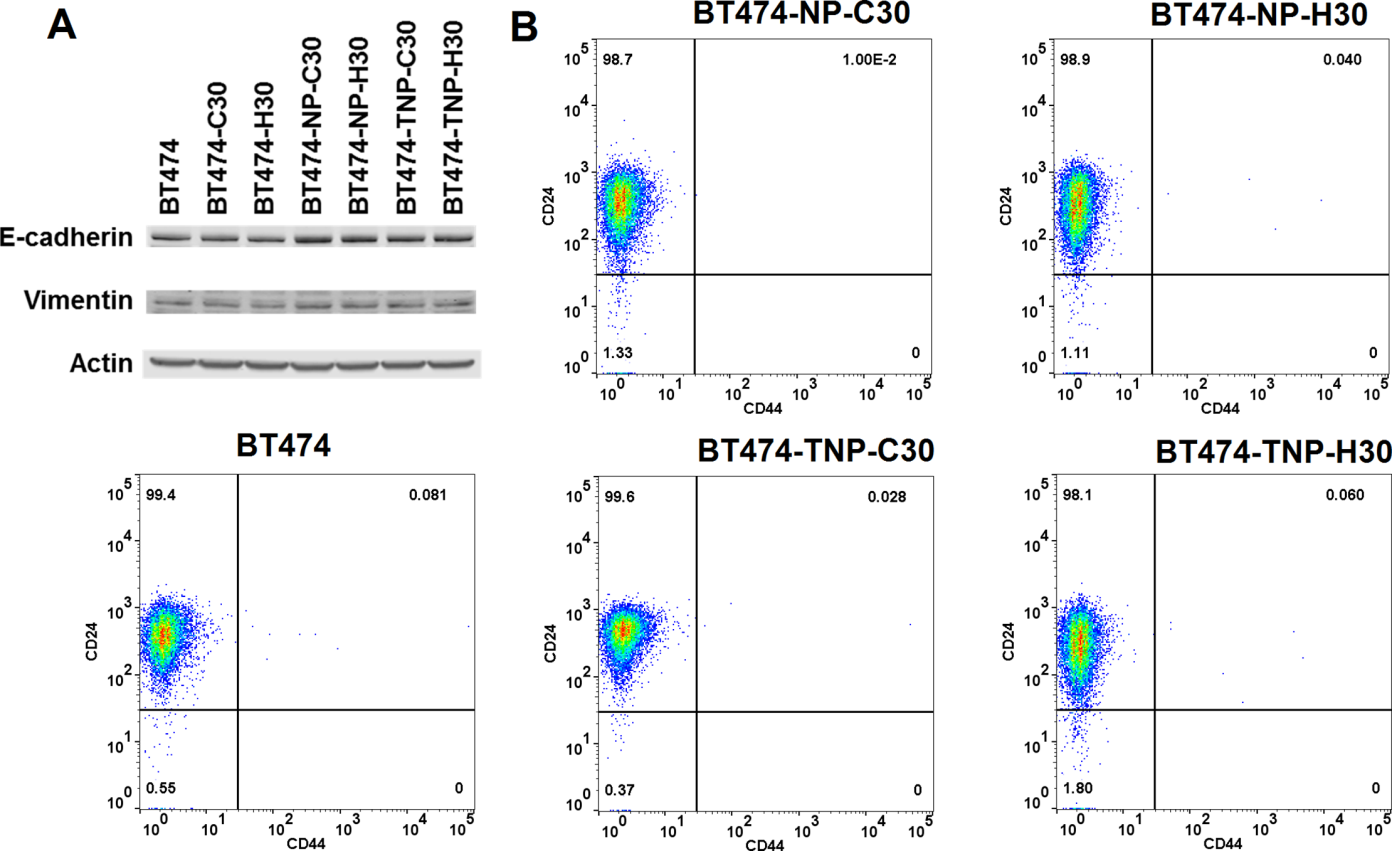
EMT and TIC characteristics of long-term HER2 siRNA treated cells. Western blot showed no concurrent downregulation of E-cadherin and upregulation of vimentin in BT474 derivatives compared to parental BT474, indicating no evidence of epithelial to mesenchymal transition **(A)**. Flow cytometry was used to determine the surface expression of CD24 and CD44 among the BT474 derivatives. There was no enrichment of tumor initiating cells (CD24-/CD44+) in the long-term treated cells **(B)**.

One mechanism by which cancer cells can develop drug resistance is through epithelial to mesenchymal transition (EMT). Cells undergoing EMT typically lose cell adhesion and become more motile, which are precursors to metastasis [40]. These changes are reflected by a decrease in the expression of E-cadherin and an increase in the expression of vimentin. Western blot from cell lysates showed that there was no concurrent downregulation of E-cadherin and upregulation of vimentin among the BT474 derivatives (Fig 2A). We next looked at the surface expression of CD24 and CD44 by flow cytometry, in which the CD24-/CD44+ population is indicative of potential tumor initiating cells (TIC) with invasive and drug resistance capabilities [41]. The parental BT474 cells were almost exclusively CD24+/CD44-, which is characteristic of luminal breast cancer. Following long-term HER2 siRNA treatment, the population distribution remains largely the same, without any detectable emergence of a CD24-/CD44+ population (Fig 2B), suggesting no enrichment of tumor initiating cells.

### Long-term HER2 siRNA treated cells remained sensitive to HER2 siRNA, trastuzumab and lapatinib

**Fig 3:**
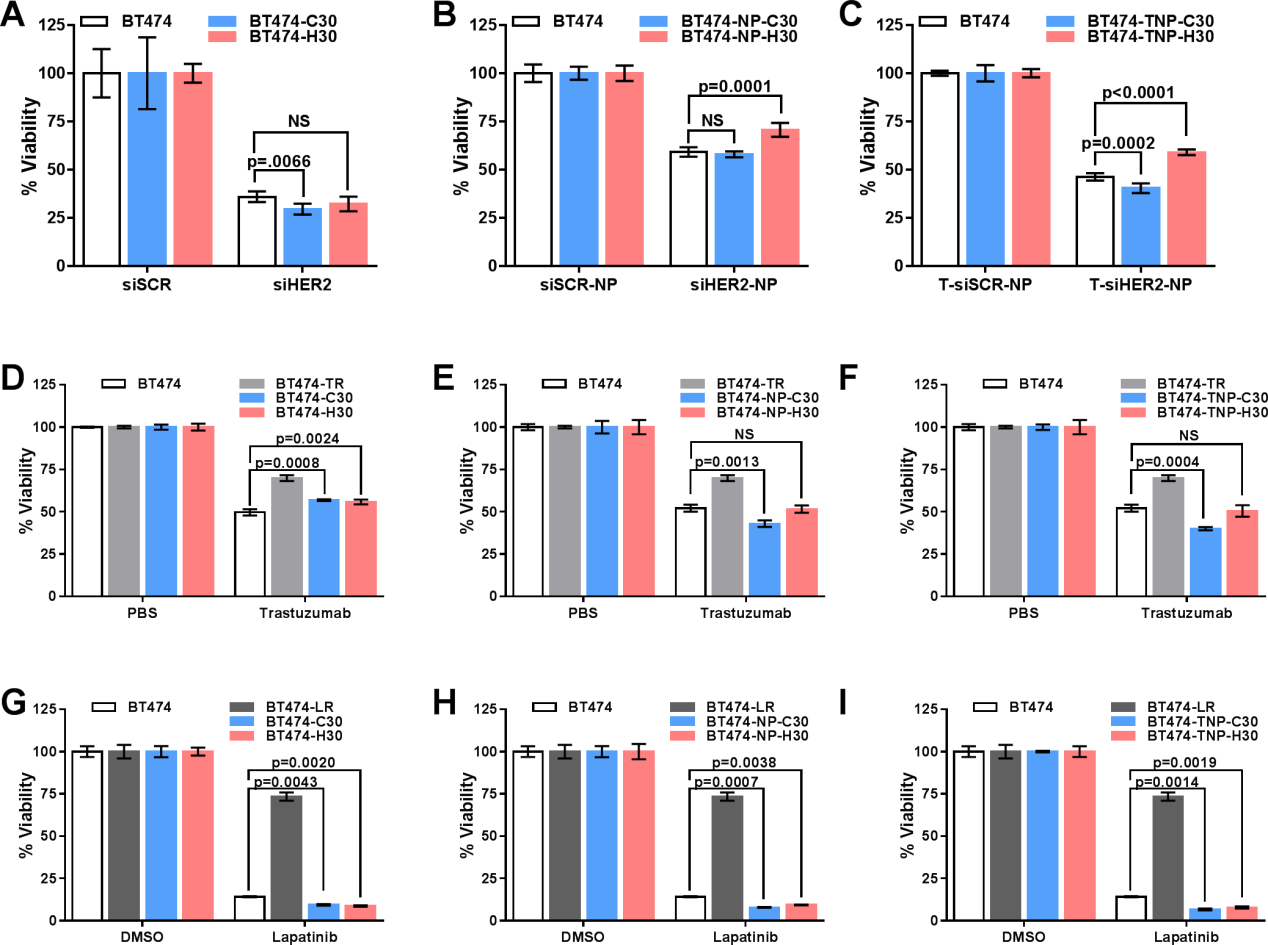
Cell viability of BT474 derivatives when challenged with their respective long-term treatment regimens. Long-term siHER2 treated BT474 derivatives were challenged with HER2 siRNA delivered using either DharmaFECT **(A)**, nanoparticles (NP) **(B)**, or trastuzumab conjugated nanoparticles (TNP) **(C)**. Their response to trastuzumab **(D–F)** or lapatinib **(G–I)** was compared to those of the resistant derivatives BT474-TR and BT474-LR.

**Fig 4:**
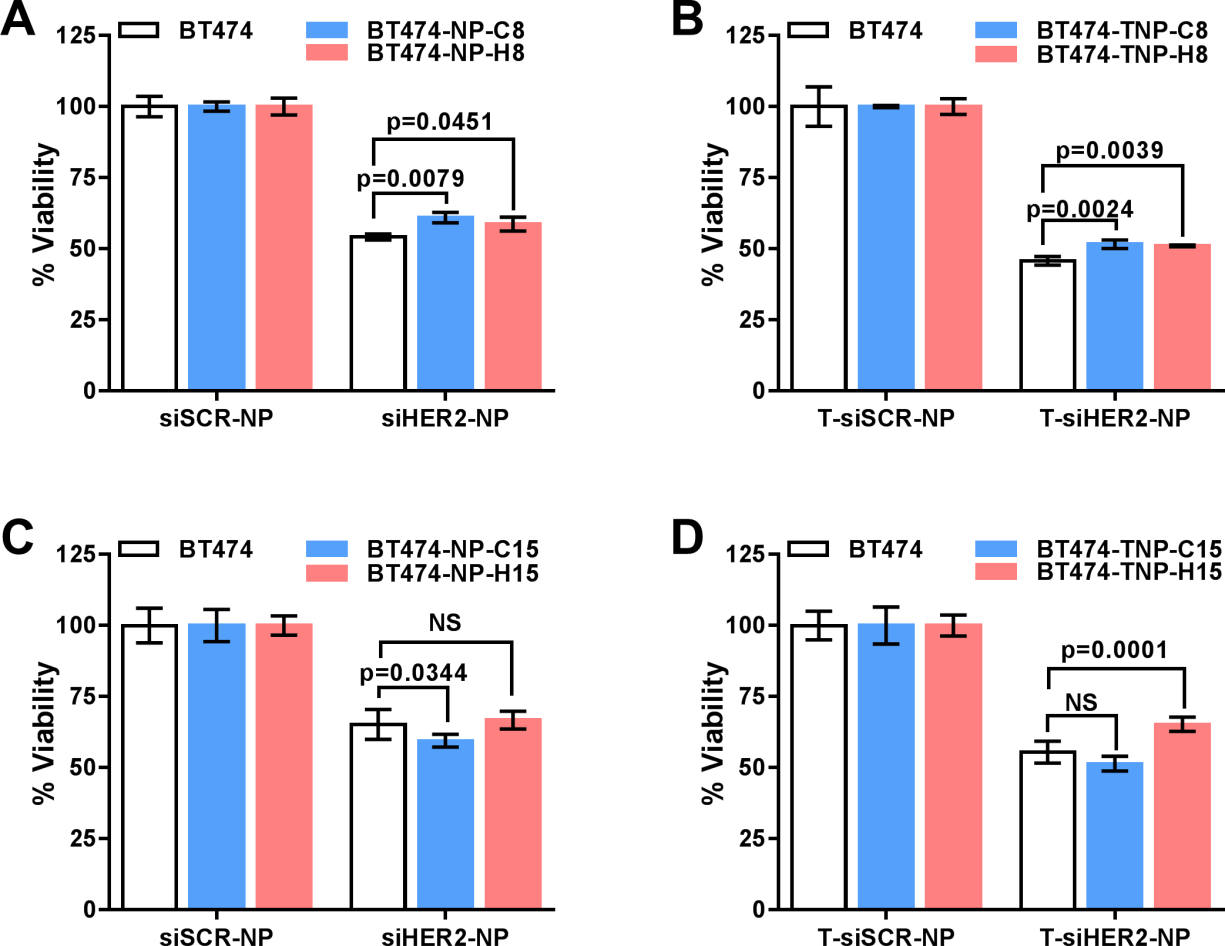
Cell viability of BT474 derivatives at earlier time points during repeated HER2 siRNA treatment. BT474 derivatives were challenged with HER2 siRNA delivered using nanoparticles **(A and C)**, or trastuzumab conjugated nanoparticles **(B and D)** after 8 weeks **(A and B)** or 15 weeks **(C and D)** of treatment.

Next, we challenged the BT474 derivatives with the same materials that they received during the 30-week treatment and assessed their responses by measuring cell viability. The response of BT474-H30 was nearly identical to that of BT474-C30 or parental cells (Fig 3A), while a reduced response was observed in those treated with HER2 siRNA delivered using nanoparticles with trastuzumab (T-siHER2-NP) or without trastuzumab (siHER2-NP) (Fig 3B and 3C). For T-siHER2-NP treated cells, the reduced response was not observed after 8 weeks of treatment but appeared after 15 weeks (Fig 4B and 4D) and remained the same from that point on. For siHER2-NP treated cells, the reduced response was not observed after 8 and 15 weeks of treatment (Fig 4A and 4C) but appeared after 30 weeks (Fig 3B). The greater reduction in response in the T-siHER2-NP group may be partially explained by phenotypic change (slower growth rate) shown in Fig 1.

We then evaluated the response of BT474 treated for 7 months (30 weeks) with HER2 siRNA in comparison to those treated for 6 months with trastuzumab and lapatinib, BT474-TR (Fig 3D–3F) and BT474-LR (Fig 3G–3I), respectively. While BT474-TR and BT474-LR were resistant to trastuzumab and lapatinib, respectively, all cells treated long-term with HER2 siRNA (delivered by DharmaFECT, NP, or TNP) did not show resistance to either drug. Furthermore, while cells became resistant to long-term treatment of free trastuzumab, they were not resistant to trastuzumab on the nanoparticles (see BT474-TR vs. BT474-TNP-C30, Fig 3F). In short, there was minimal change in terms of how long-term HER2 siRNA treated cells responded to renewed HER2 silencing or inhibition using lapatinib or trastuzumab. Thus, silencing HER2 did not induce adaptive responses that would have rendered the cells resistant to repeated HER2 silencing (with siRNA) or inhibition, at least not to the same degree and within the same time frame as in BT474-LR or BT474-TR.

### HER2 ablation prevented reactivation of HER2 signaling

**Fig 5:**
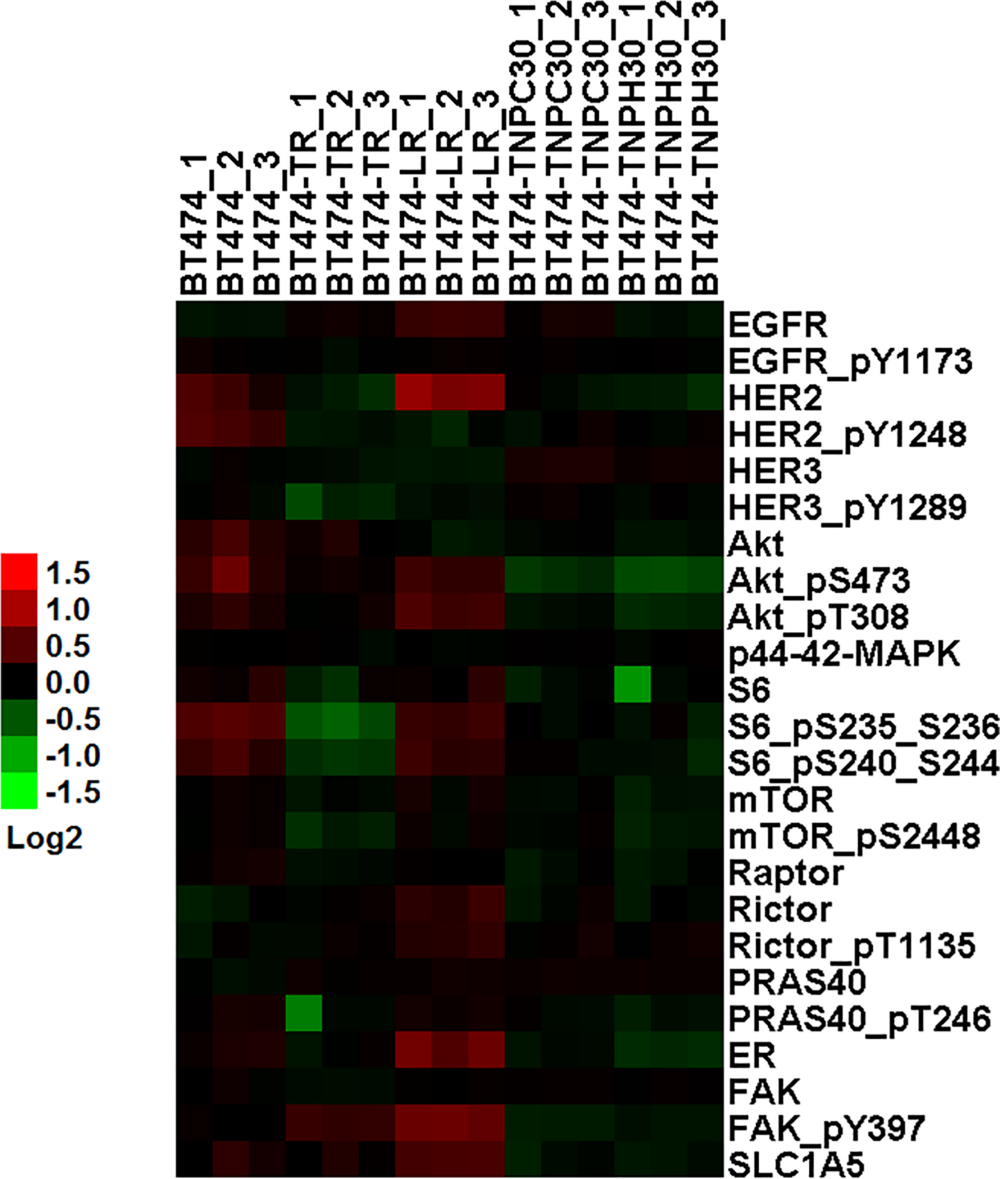
Heat map of selected genes that were differentially expressed as determined by RPPA. Proteins from BT474 derivatives while under their corresponding long-term treatment agents were spotted onto reverse phase protein microarrays and probed using a panel of antibodies. Data are presented in triplicate, with each data point representing the expression of a given protein from a single replicate. Color gradient represents protein expression levels (log base 2). The original full panel raw data are available as S1 Dataset.

To explore how HER2 siRNA treated cells differ in adaptation to the selective environment when compared to BT474-TR and BT474-LR, we looked at changes in protein expression and phosphorylation using reverse phase protein arrays. Data are shown in Fig 5. The parental BT474 served as a baseline (no treatment). BT474-TR, BT474-LR, BT474-TNP-H30 and BT474-TNP-C30 were sampled while under treatment of trastuzumab, lapatinib, HER2 siRNA and scrambled siRNA delivered with TNP, respectively. In the lapatinib resistant BT474-LR, we observed an increase in the expression of ER and HER2, in agreement with compensation through ER and reactivation of HER2 signaling in lapatinib resistant cells reported by Wang and colleagues [35]. Phosphorylation of AKT, ERK and S6 ribosomal protein were sustained even in the presence of lapatinib. There was a slight increase in the expression and phosphorylation of Rictor, which is a cofactor of the mTOR complex 2 that was shown to mediate resistance through phosphorylation of AKT on serine 473 [42]. Phosphorylation of focal adhesion kinase (FAK) was also increased in BT474-LR and BT474-TR, which is consistent with a previous report of its physical association with HER2 to mediate receptor clustering and crosstalk [43]. Upregulation of SLC1A5 (ASCT2), an alanine, serine, cysteine-preferring transporter 2 was observed in BT474-LR. This protein was shown to regulate glutamine uptake and promote growth of triple negative breast cancer by supporting the mTORC1 signaling [44]. These changes, however, were not present in BT474-TNP-H30, in which reduced level of HER2 signaling was observed. BT474-TNP-C30 (with scrambled siRNA) also had downregulation of HER2, which can be attributed to trastuzumab on the nanoparticles. This suggests that even at a very low dose (equivalent to 1 μg/ml trastuzumab), trastuzumab on the nanoparticles could reduce HER2 protein level via receptor internalization (along with the nanoparticles) and degradation. As a result, we did not observe higher levels of phospho-FAK in BT474-TNP-C30 as we did in BT474-TR.

## Discussion

Targeting the HER2 receptor using conventional monoclonal antibodies and small molecule inhibitors works by blocking dimerization and activation of the receptor. However, HER2 can bind many different receptors outside of the HER family and thus circumvent inhibition via receptor crosstalk and alternative signaling pathways. These adaptive changes can lead to the survival of a small population of cancer cells that persist throughout treatment, leading to the relapse of the disease. We showed that using siRNA to silence HER2 at the mRNA level, thereby halting synthesis of the HER2 protein, could prevent these survival mechanisms.

In this study, we compared the endpoints of separate populations of HER2-positive BT474 cells after receiving trastuzumab, lapatinib or HER2 siRNA for 6–7 months. In the trastuzumab or lapatinib groups, the cells became markedly resistant, with a 250 and 20 fold increase in GI50 values for each, respectively, as reported previously [36]. The HER2 siRNA treated groups, on the other hand, remained sensitive to repeated HER2 silencing, trastuzumab and lapatinib. We did not observe resistance to long-term HER2 siRNA treatment when delivered with DharmaFECT, but a 10% reduction in response was observed when delivered with nanoparticles, especially those conjugated with trastuzumab. The reduction in the response could be attributed to the slower growth rate after T-siHER2-NP treatment. During the long-term treatment, a low concentration of HER2 siRNA was used throughout the entire period because we were unable to increase the concentration over time while retaining enough cells to subculture. This further supports the conclusions that HER2 siRNA treatment was highly growth inhibitory and that the BT474 cells were unable to circumvent siRNA mediated HER2 ablation. Our data also indicated that HER2 siRNA delivered by our nanoparticles did not induce epithelial-mesenchymal transition or enrichment of tumor initiating cells.

Reactivation of HER2 signaling after long-term exposure to lapatinib was attributed to the secretion of HER family receptor ligands as well as the HER2 L755S mutation [45]. In our version of lapatinib resistant BT474-LR, we also observed higher expression of HER2 and ER. This derivative also displayed higher phosphorylation of focal adhesion kinase (FAK), which was implicated in HER2 receptor clustering with integrin β1, leading to amplified signaling [43]. Another group has shown that inhibiting FAK could improve response to trastuzumab [46]. These findings suggest that HER2-positive cancer cells can develop converging mechanisms to maintain HER2 signaling and are therefore susceptible to HER2 ablation using siRNA.

While HER2 mutations are relatively rare in the clinic, their occurrence can effectively limit the efficacy of inhibitors or antibodies, as in the case of the L755S mutation. One major advantage of using siRNA as a therapeutic on a modular delivery platform such as our nanoparticles is that siRNA can be quickly redesigned and validated should a mutation render the original version ineffective, although new clinical trials would still be required.

Another benefit of siRNA therapeutics is the ability to target genes for which there are significant technical challenges in developing an effective small molecule inhibitor, as in the case of KRAS. A recently completed phase 1 trial delivered KRAS siRNA intratumorally in combination with chemotherapy in advanced pancreatic cancer [47]. The regimen was well-tolerated and showed promising efficacy, prompting the initiation of a phase 2 trial.

Systemic delivery of siRNA requires effective nanoparticle carriers to achieve promising response in tumors not amendable for local delivery. Our nanoparticle platform has been highly optimized and validated in various mouse models of human cancers [36, 37, 48] and geared toward clinical trials. In this study, we reported for the first time the effect of long-term siRNA treatment in cancer. We showed that HER2-positive cancer cells are less likely to develop resistance to HER2 siRNA than to trastuzumab or lapatinib over the same period of treatment in vitro. From a mechanistic standpoint, the ablation of HER2 protein is more effective than blocking HER2 activation. We have previously shown that silencing HER2 could also overcome trastuzumab resistance in vivo [36]. However, further investigation is needed to determine whether HER2-positive tumors can develop resistance to T-siHER2-NP after repeated administration in vivo. A side by side comparison with trastuzumab and lapatinib in vivo is also needed. In addition, the use of immuno-competent mice to compare the impact of immune mediated killing of cancer following trastuzumab treatment versus HER2 knockdown by siRNA is also of interest. Overall, our siRNA therapeutic has promising clinical values and warrants further pre-clinical and clinical investigation.

## Supporting information

S1 Dataset Full panel RPPA raw data. (XLSX)
